# Control Deficits, Conditioning Factors, and Playing through Pain and Injury among Iranian Professional Soccer Players

**DOI:** 10.3390/ijerph18073387

**Published:** 2021-03-25

**Authors:** Saeed Kabiri, Jaeyong Choi, Seyyedeh Masoomeh (Shamila) Shadmanfaat, Julak Lee

**Affiliations:** 1 Department of Humanities and Social Sciences, University of Mazandaran, Babolsar 4741613534, Iran; s.kabiri89@gmail.com; 2 Department of Security Studies and Criminal Justice, Angelo State University, San Angelo, TX 76901, USA; jaeyong.choi@angelo.edu; 3 Department of Literature and Humanities, University of Guilan, Rasht 4199613776, Iran; shamila.shadmanfaat@gmail.com; 4 Department of Industrial Security, Chung-Ang University, Seoul 06974, Korea

**Keywords:** control balance theory, playing through pain and injury, professional athletes

## Abstract

Playing through pain and injury is a common and accepted behavior in the athletic realm. The purpose of this research was to apply Tittle’s control balance theory to explain why athletes engage in playing through pain and injury despite its risky nature. We hypothesized that playing through pain and injury is a form of submission described by Tittle and that it can be predicted by the concept of control deficit. To this end, we collected and used data from a sample of 410 professional soccer players from Guilan province, Iran, and tested several propositions derived from control balance theory. Hierarchical linear regression was used to analyze the data. The study findings demonstrate that players with more control deficits are more likely to play through pain and injury. This relationship is conditioned by self-control, opportunity, motivation, perceived benefits, and provocations. For example, the relationship between control deficit and playing through pain and injury is stronger for those with lower self-control. Our findings support the utility of control balance theory in explaining an act of submission (i.e., playing through pain and injury).

## 1. Introduction

Only a few athletes can meet physical performance standards in professional sports and obtain high athletic status. Some athletes attempt to improve their sports performance by relying on risky behaviors, such as playing through pain and injury [1]. Other athletes choose to play with injuries suppressing their negative emotions and feelings because they fear portrayal as weaklings who cannot compete at a professional level [2,3,4]. Still, other athletes play with pain and injury because they believe that these challenges are necessary evils on the road to success and acceptance by coaches and teammates [5,6]. In short, scholars have viewed risk-taking behavior as an inevitable part of the sports world that has different origins, such as social desirability, the sense of masculinity, sports identity confirmation, and over-conformity [5,7,8,9].

The current study investigates the issue of playing through pain and injury by applying Tittle’s [10,11] control balance theory. Control balance theory was created to explain various types of deviant conduct. We view playing through pain and injury as deviant behavior because it is inconsistent with the social norm. Given that professional athletes are subject to various types of social, structural, and other kinds of influences (e.g., coaches, teammates, or fans), and that these conditions can stimulate athletes’ actions, Tittle’s insight regarding control can be helpful in understanding athletes’ playing through pain and injury. Specifically, we posit that the ratio of the amount of control that athletes exercise to the amount of control they are subject to can influence the likelihood of playing through pain and injury based on control balance theory.

Using data drawn from professional soccer players in Iran, we assessed whether control deficit is a significant predictor of athletes’ tendency to play through pain and injury. Additionally, interaction analyses were performed to explore variables that condition the relationship between control deficit and the behavioral tendency to play through pain and injury. Doing so not only sheds light on the phenomena involving athletes’ playing through pain and injury, but serves the broader purpose of identifying the potential theoretical framework useful in establishing policy implications.

## 2. Literature Review

### 2.1. Tittle’s Control Balance Theory

Tittle’s [10,11] control balance theory proposed that all people have a certain amount of control that they can exert and a certain amount of control to which they are subject. According to this theory, control involves the power to change or manipulate other people’s actions or environments. Tittle [11] defined control ratio as “the amount of control to which an individual is subject, relative to the amount of control he or she can exercise”. When the relative level of control is in balance, deviance is unlikely to occur. Conversely, when the control ratio is imbalanced, individuals can be motivated to engage in deviant behavior to restore control balance. This has two types: control deficit and control surplus. People suffer from a control deficit when they are under a greater amount of control relative to the amount of control they can exert. Others experience a control surplus because they can exert more control over others than the control to which they are subject. In this study, we focused on control deficits among soccer players because Tittle’s [11] original statement of control balance theory suggests that control deficits predict repressive forms of deviance where the individuals attempt to restore some of their control deficits. Repressive forms of deviance include acts of submission, and playing despite pain and injury can be viewed as acts of submission to please coaches, team members, and fans at the expense of the athlete’s health.

Although control imbalance can cause deviance, Tittle [11,12] hypothesized that the impact of control imbalance on deviance could depend on various factors. First, Tittle identified the sources of predisposition to deviant behavior; individuals’ tendency to strive for autonomy, blockage from desired goals, and control imbalance. The convergence of these three factors can result in the predisposition to motivation for deviance. Second, the development of motivation for deviance involves the conditions: (a) individuals recognize their control imbalance and believe that involvement in deviant behavior can result in a return to balance; (b) a negative emotion is triggered by situational provocation (e.g., humiliating situations).

Another vital discussion involves the type of control ratio. An individual’s general control ratio can be stable, reflecting their social positions, statuses, and personalities [11]. However, one’s control ratio may vary depending on the specific domain “as the individual confronts different social and physical entities and as different personal and situational characteristics come into play” (p. 148). For instance, people may have different levels of control ratios specific to life domains, such as family, peers, or work, which comprise a global control ratio. Because individuals may have a unique domain associated with a specific control ratio, Piquero and Hickman [13] recommended that “when measuring the control balance, ratio, it is important to include domains of life that are relevant to the sample under investigation” (p. 329).

The tenets of Tittle’s [10,11] control balance theory have been tested through the use of self-reported data on assault [14,15,16], theft [14,16,17], academic cheating [17], alcohol and drug consumption [14], unhealthy dieting [18], victimization [19], stalking victimization [20], and police deviance [21]. Control balance theory has also been tested with different sample types [15,17,19,22,23,24,25], including homeless youth [26,27], juveniles [28], and police officers [21]. Across these studies, the utility of control balance theory has been generally supported by the data [29], especially regarding the core proposition that control imbalance can motivate people to engage in deviant behavior to restore a sense of control. However, the efficacy of control balance theory has not been extensively tested in Asian sample populations [30], and this theory has not been utilized to examine and account for some athletes’ tendency to play despite injuries and pain [31,32].

### 2.2. Control Balance in the Sports Domain and Playing Through Pain and Injury

Research has shown that athletes often consider pain and injury a routine part of sports performance and disregard sports injury risk [6,33,34]. Many qualitative studies have been conducted to explore why athletes continue to participate in sports despite the risk of injury and pain. Charlesworth and Young’s [33] study showed that both men and women athletes often hide, deny, and suppress pain and injuries because they believe that expressing concerns about pain and injury can weaken their sports identity in the eyes of others (i.e., being labeled as weak or negative). Similarly, Sinden [34] observed that athletes suppress negative and unpleasant emotions because play, despite the development of health problems, has been normalized in their sports environment and because athletes monitor and coerce each other to follow the established norms. In this context, athletes gradually learn to accept the risk of pain and injuries and accustom themselves to risky behaviors such as the excessive overweight control associated with an eating disorder, taking untested performance-enhancing drugs (PEDs), over-training, and playing through pain and injury. Sinden [5] argued that athletes put aside their fear and anxiety involving injury to avoid being judged negatively by their teammates and coaches. Athletes also try to exhibit a hard and tough personality to their significant others by suppressing their emotions. The internalization of established norms—fearlessness, risk-taking, and denying negative emotions—causes athletes to expose their bodies to different forms of risky behaviors. In short, previous research tended to focus on socialization to pain and injury (e.g., sports ethic) among athletes by illuminating how the sports culture advances the notion of playing through pain and injury (see also [35,36]).

However, these studies have failed to consider that athletes are both subjects of socialization and agents who exercise control over their lives. Tittle’s [10,11] control balance theory recognized that individuals could influence things around them. In this vein, the control balance theory can apply to understand athletes’ behavioral patterns [30]. Specifically, athletes may perceive that they have lost their control in the sports domain in several ways: (a) losing their spots to other athletes, (b) being treated as untrustworthy by coaches, team members, and fans, or (c) poor sports performance. These athletes may try to gain more control over the sports domain and balance the control ratio. If an athlete’s control ratio is unbalanced, he or she may engage in deviant behavior to change their control imbalance, such as doping, overtraining, excessive diet, or playing while injured [5,9,30].

According to the control balance theory, individuals’ level of control—compared to the level of control to which they are subject—affects the likelihood of deviant behavior. In the sports setting, the relationship between control imbalance playing through pain and injury depends on an athlete’s control ratios in his/her sports domain and other life domains. People can experience different levels of control ratios depending on the life domain. For example, athletes may have a control surplus at home while having a control deficit in their sports domain. The control deficit in their sports setting may be attributed to strong team competitors, sports injuries, coaches’ distrust, and poor performance. Once athletes recognize this lack of control, they may become motivated to extend control (expand his/her power in the sports setting) through playing through pain and injury. This control balancing process may be intended to improve their sports performance (more opportunities can be provided to participate in sports competitions), gain coaches’ and teammates’ trust (athletes can demonstrate their commitment to the game), obtain stable positions in their sports teams (those athletes injured may be more disposable), and attain sports success [6,36].

When discussing the revised version of his control balance theory, Tittle [10] elaborated on the role of self-control in the relationship between control imbalance and deviance. He argued that self-control is “the personal ability to restrain his/her desire to act immediately to gratify whatever emotional desires (such as overcoming humiliation) that might arise” (p. 415). He hypothesized that those with low self-control tend to engage in deviant behavior that requires personal contact because they seek immediate gratification. Conversely, those with high self-control were hypothesized to be more likely to engage in impersonal deviant behavior because they can resist the urge for immediate action. Given that playing through pain and injury is an impersonal risky behavior, athletes with high self-control may be more likely to play through pain and injury.

According to the control balance theory, situational provocations can remind individuals of their control imbalance (e.g., verbal insults or degrading comments) [30]. If coaches treat athletes in humiliating or degrading ways, they are more likely to play through pain and injury. Another important concept in control balance theory, motivation, involves an internal drive to exert more control over life domains and attain freedom and independence [11]. Athletes may be motivated to prove themselves to coaches, fans, and teammates and increase the amount of control they can exercise in the sports setting. Additionally, athletes with higher motivation may be more likely to be involved in deviant behaviors when recognizing a control deficit [21].

Opportunity concerns a necessary situational condition for individuals to engage in deviance [11]. Opportunity can serve as a conditioning factor, influencing the impact of control imbalance on deviance. Accordingly, athletes with more opportunities are more likely to play through pain and injury when they perceive control deficit. Finally, perceived benefit refers to rewards and costs associated with deviant and risky behaviors. Athletes may perceive the benefits of playing through pain and injury, such as improving their athletic performance and strengthening their team positions. Those with higher perceived benefits may be more involved in playing through pain and injury when they are aware of their control deficit.

## 3. Current Study

**Figure 1:**
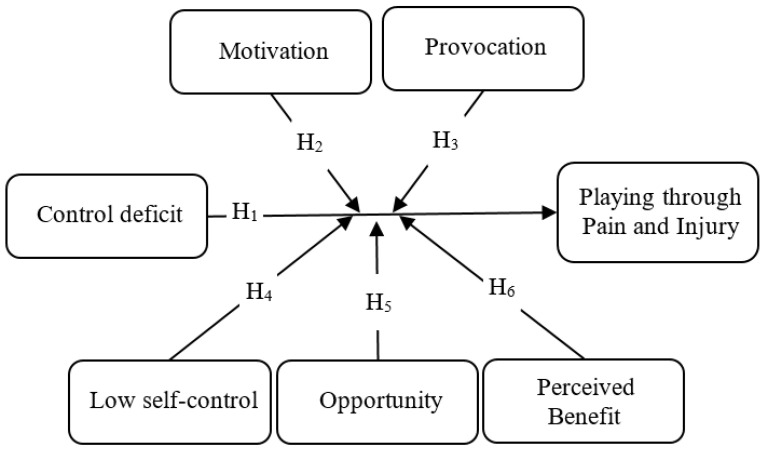
The conceptual model of the relationship between control balance and playing through pain and injury.

The current study tested the utility of Tittle’s [10,11] control balance theory in the examination of playing through pain and injury. According to control balance theory, athletes can suffer from control deficits (in different situations, such as strong competitors on a team, sports injuries, or poor performance). These challenges are often beyond their control and can make them worry about losing their source of income, social status, and desired future goals [3,5]. Athletes may be motivated to restore their control balance through risky behavior, such as playing through pain and injury. Playing despite them may be an attractive option for athletes to regain their control because it can improve their sports performance, gain coaches’ and teammates’ trust, and obtain stable positions on their sports teams [6,7]. Therefore, we examine whether the control deficit has a significant effect on playing through pain and injury. Simultaneously, the theoretical interactions between control balance and other major concepts are explored. The study’s conceptual model is presented in Figure 1.

Hypothesis 1–6 for this study are as follows:

**Hypothesis** **1:** *The control balance (deficit) ratio will be significantly and positively associated with playing through pain and injury among Iranian soccer players.*

**Hypothesis** **2:** *Motivation will strengthen the relationship between control balance (deficit) ratio and playing through pain and injury.*

**Hypothesis** **3:** *Provocation will strengthen the relationship between control balance (deficit) ratio and playing through pain and injury.*

**Hypothesis** **4:** *Low self-control will strengthen the relationship between control balance (deficit) ratio and playing through pain and injury.*

**Hypothesis** **5:** *Opportunity will strengthen the relationship between control balance (deficit) ratio and playing through pain and injury.*

**Hypothesis** **6:** *Perceived benefit will strengthen the relationship between control balance (deficit) ratio and playing through pain and injury.*

## 4. Data and Methods

### 4.1. Data

A cross-sectional survey was used for this study to apply Tittle’s [11] control balance theory to playing through pain and injury. We collected self-report data from 410 Iranian professional soccer players. We obtained the list of professional soccer athletes from the Physical Education Organization of Guilan province. The target population of the study consisted of 990 professional soccer players (840 male players and 150 female players) of 32 professional men’s and women’s soccer teams (28 male teams and 5 female teams) that were active in Iran’s professional leagues (first division, second division, third division, and Guilan premier league). The obtained list from the Guilan Education department served as the sampling frame for the present study. Potential participants were randomly selected based on the athlete list. In the four weeks of the soccer leagues 2018–2019, we contacted the soccer teams’ coaches to schedule the visit and attended the training sessions. We explained the research purpose to soccer players and obtained their consent to participate in the research. The questionnaires were distributed in the large meeting space before training sessions. The sampled athletes provided voluntary consent to fulfill the institutional review board (IRB) requirements. The IRB from the University of Mazandaran approved this research, and the registration code for this research proposal is 1339159. The final data were registered in the Iranian Research Institute for Information Science and Technology (IranDoc).

We calculated the adequate effect size using the G*Power software with power (1 − β) set at 0.95 and α = 0.05, two-tailed. To detect the medium effect size (*f*² = 0.15), a total sample of 146 would be needed with six predictors. To ensure that we obtain the appropriate sample size, we collected additional questionnaires. The research team distributed 448 questionnaires (318 questionnaires among males and 130 questionnaires among females), and data from 410 questionnaires (294 questionnaires from males and 116 questionnaires from females) remained after excluding missing data. All the questionnaires used in the research have been completely answered. The researchers have dropped the questionnaires that had missing values. The response rate was 91.52%.

### 4.2. Measures

#### 4.2.1. Dependent Variable: Playing through Pain and Injury

Playing in the face of pain and injury involves an athlete’s behavioral pattern of playing a sport even when it is likely to result in pain and injury [7,37]. We used three items to measure this behavior, employed in a previous publication [37]. Specifically, participants were asked to indicate (a) whether they had played through pain and injury that had threatened their overall health, (b) whether they had deliberately hidden the pain and injury that could have harmed their health and continued to play, and (c) whether they had ever endured psychological strain (fear, anxiety, distress, rumination) associated with an anticipated injury, but continued to practice and play. The question was answered using a 5-point Likert scale from 1 = almost never to 5 = almost always.

#### 4.2.2. Independent Variables

General and sports control balance ratio: the control balance ratio was measured by adopting Hughes et al.’s and Piquero and Hickman’s methods [13,14]. However, some important modifications were made to that scale because of our focus on the sports-oriented sample. Several control-exercised measures specific to the sports field were added. We assessed several domains of control exercised, summed them up to represent their respective domains, and then calculated the general control (deficit) and sports control (deficit) ratios. Hughes, Antonaccio and Botchkovar [14] provided the following definition of control to help participants understand control balance survey items: “Control means having the ability to influence things around you, to make things happen the way you want them to, to accomplish your goals, to do whatever you wish, to make things easier or harder for other people, to determine your own fate” (p. 960). Our participants read the definition before responding to survey items.

Following Piquero and Hickman’s [13] measure of control balance ratio, we asked respondents to report the level of control exercised over seven life domains (e.g., friendship, family relationships, and physical body). We also used the sports control balance measure from Kabiri, Shadmanfaat, and Donner [30]. We asked respondents to report how much control they thought they could exercise over seven sports domains (e.g., coaches, relationships with fellow athletes, and sports performance). Each item was reported using a 4-point scale, from 1 (no control) to 4 (total control).

Low self-control: consistent with published papers testing control balance theory [13,17,18,21], the 6-item scale derived from Grasmick, et al. [38] was used to measure low self-control. It included these items: 

“I often act on the spur of the moment without stopping to think,”; “I often try to avoid things that I know will be difficult,”; “I lose my temper pretty easily,”; “When I am really angry, other people better stay away from me,”; “I often take a risk just for the fun of it,”; “Sometimes I find it exciting to do things that are dangerous.” Respondents rated their agreement with each statement using a 5-point Likert type scale, from 1 (strongly disagree) to 5 (strongly agree), and the responses were summed to represent the level of low self-control.

Provocations: a four-item self-report scale was used to measure provocation [30]. Respondents were asked about their level of agreement with the following statements: “My position on the team is unstable,”; “I have strong competitors in the team,”; “My position in the team is threatened every day,”; and “I am always afraid of losing my place on the team.” Each item was reported using a 5-point Likert scale, from 1 (strongly disagree) to 5 (strongly agree).

Motivation: motivation was measured by a four-item self-report scale. Our motivation measure was designed to be specific to play through pain and injury, adapting measures of motivation that were used in previous research [30]. Respondents were asked about their level of agreement with the following statements: “I should tolerate pain and injury to prove myself to the coaches,”; “I should tolerate pain and injury to prove myself to the fans,”; “I should tolerate pain and injury to get more time to play,”; and “I should train myself harder (giving my body more work or stress than it can handle) to get more time to play.” The responses ranged from 1 (strongly disagree) to 5 (strongly agree).

Opportunity: according to Tittle [11], there should be an opportunity for deviant behavior to occur. To measure the opportunity to play with pain and injury, we used the beliefs of sports community members regarding pain and injury. We hypothesized that if coaches, teammates, and fans had positive beliefs about pain and injury, the athletes would have more opportunities to play with pain and injury. Specifically, the following three items were used: “The people around me (coaches, teammates, fans) believe that athletes who endure pain and play deserve our respect,”; “The people around me (coaches, teammates, fans) believe that athletes who care about their team will try to play with injuries and pain,”; and “The people around me (coaches, teammates, fans) believe that athletes should ‘tough it out’ with an injury or pain today and not worry about the effects tomorrow.” The responses ranged from 1 (strongly disagree) to 5 (strongly agree).

Perceived benefit: a four-item self-report scale was created to measure the perceived benefit of pain and injury by adapting the Piquero and Piquero [23] measure of perceived benefit. Respondents were asked to respond to the following questions: “How much would it advance your sports career if you accepted and tolerated pain and injury?”; “How much would it advance your sports career if you played with pain and injury?”; “What is the chance that the coaches would exclude you if you didn’t play with pain and injury?”; “What is the chance you personally would be rejected by the fans if you didn’t play with pain and injury?”; “What is the chance you personally would be rejected by the fellow athletes if you didn’t play with pain and injury?” The responses ranged from 1 (very low) to 5 (very high).

#### 4.2.3. Validity and Reliability of Measurement Instruments

**Figure 2:**
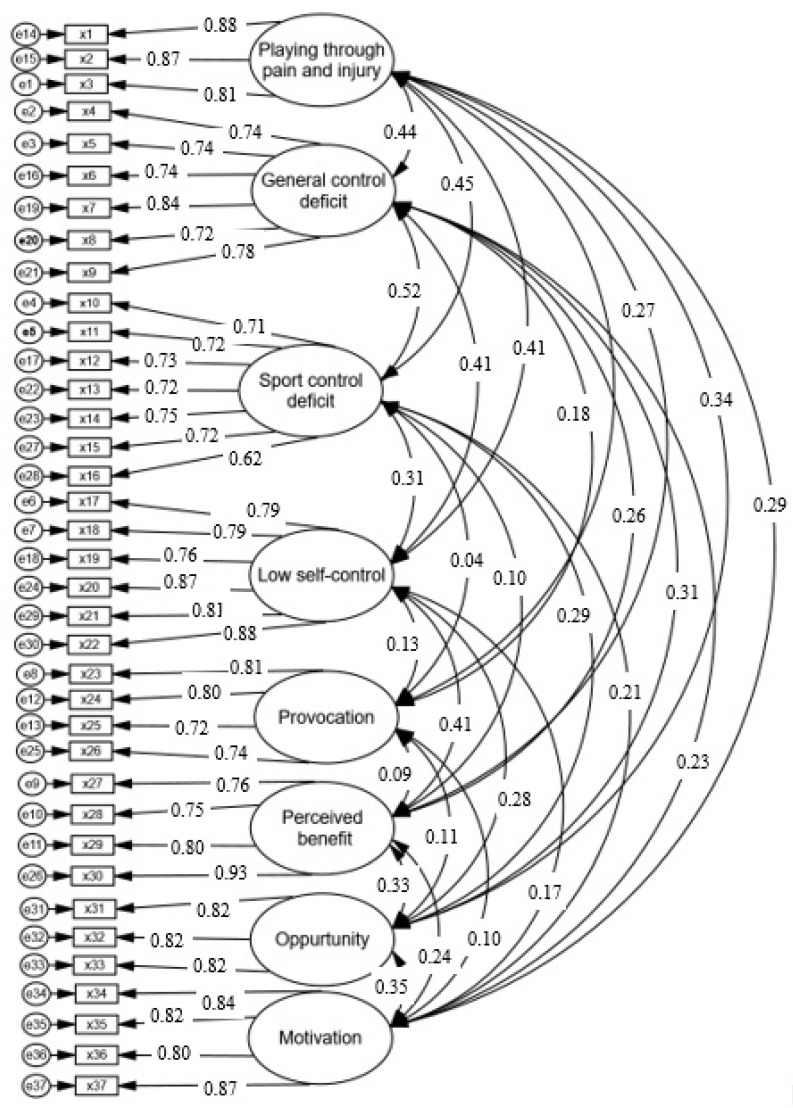
Confirmatory factor analysis model.

All scales exhibited high internal consistency (α > 0.70; Composite Reliability (CR) > 0.70) (see Table 1). We also assessed their discriminant validity by estimating the average variance shared between a construct and its measures (AVE). AVE indices were higher than 0.50, meet the recommendation by Fornell and Larcker [39]. Finally, we performed a first-order confirmatory factor analysis (CFA) for these scales; factor loadings for all of the items were greater than 0.50 (See Figure 2).

Additionally, the results from CFA revealed good fit indices for all scales, where the summary statistics (i.e., Relative chi-square (1.422), Comparative Fit Index (0.971), Standardized Root Mean Square Residual (0.037), and Root Mean Square Error of Approximation (0.032)) are better than critical values [40].

### 4.3. Method of Analysis

In order to evaluate the utility of control balance theory in explaining playing through pain and injury, hierarchical linear regression was implemented. We examined the assumptions of multiple regression were checked. We found that there was a linear relationship between the independent variable(s) and the dependent variable. No issue of multicollinearity was observed when we examined variance inflation factor scores. We confirmed the normal distribution of error terms. Hayes’ PROCESS version 3.1 macro in SPSS (IBM Corp., Armonk, NY, USA) was used to test the regression lines’ slope in moderator models.

## 5. Results

Demographic findings of this study indicated that 71.1% of athlete respondents were men, and 28.3% were women. Of them, 25.1% were under 20 years of age, 24.9% were between 20 and 25 years old, 27.3% were between 26 and 30 years old, and 22.7% were over 30 years old. Moreover, 55.6% of athletes were single, and 44.4% of them were married. Furthermore, 10.5% of respondents were high school students; 42.9% of respondents were high school graduates; 22.4% had an associate’s degree; 17.8% had a bachelor’s degree; and 6.3% had master’s or higher degrees. Moreover, 34.4% of respondents had less than five years of sport participation experience, 28.8% of them ranged from 5 to 10 years, 25.4% of them ranged from 11 to 15 years, and 11.5% had more than 15 years of sport participation experience.

Table 2 represents the correlations between playing through pain and injury and other variables. As the table indicates, there are strong correlations between playing through pain and injury and control balance theory components.

Table 3 shows findings from the various ordinary least squares regression analyses. Before implementing the regression models, we mean-centered variables prior to computing product terms (to serve as a moderator term) to reduce multicollinearity in a regression model. We used hierarchical multiple regression to evaluate the interaction effects. As a result, the following 14 regression models briefly examine the interaction terms of variables derived from control balance theory. Models 1 and 2 show the moderation effect of low self-control; Models 3 and 4 indicate the moderation effect of provocation; Models 5 and 6 indicate the moderation effect of motivation; Models 7 and 8 show the moderation effect of opportunity; Models 9 and 10 show the moderation effect of perceived benefits; Models 11 and 12 involve the moderating effects of all variables in a total regression model.

**Figure 3:**
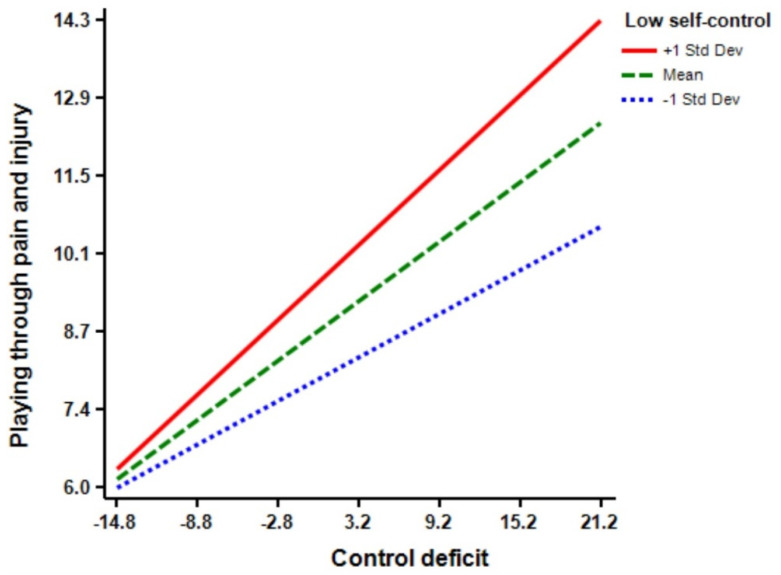
Low self-control by control balance deficit interaction.

**Figure 4:**
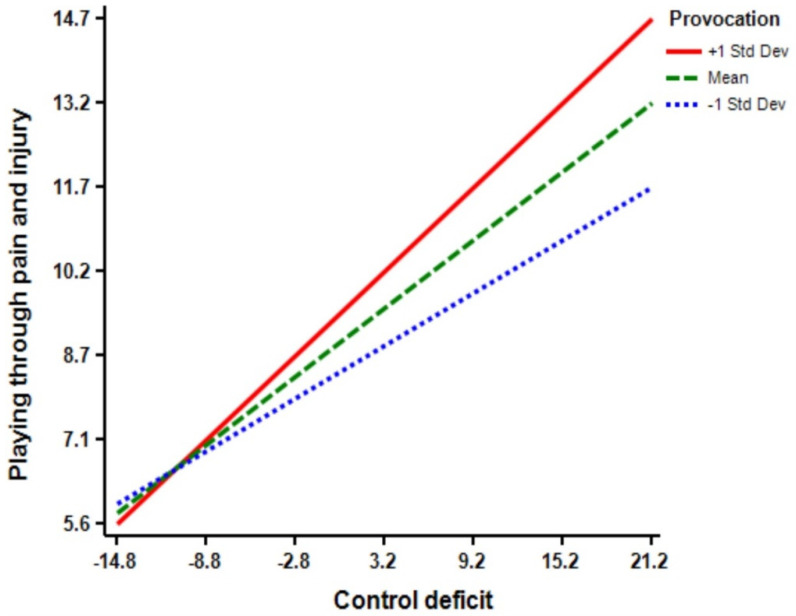
Provocation by control balance deficit interaction.

According to Table 3, the regression model (Model 1) shows that control deficit (Β = 0.38 **, *p* < 0.01) and low self-control (Β = 0.24 **, *p* < 0.01) have significant effects on athletes’ playing through pain and injury. Moreover, the regression model (Model 2) indicates that the interaction terms of control deficit × low self-control have a significant effect on playing through pain and injury (Β = 0.11 **, *p* < 0.01). The inclusion of control deficit × low self-control interaction in playing through pain and injury increased the predictive power of the main effect by 0.01 percent (R squared change = 0.01, F Change = 6.92 **, *p* < 0.01) from 0.27 percent to 0.28 percent (See Figure 3). Furthermore, the regression model (Model 3) reveals that provocation (Β = 0.14 **, *p* < 0.01) has a significant effect on athletes’ playing through pain and injury. Moreover, the regression model (Model 4) shows that the interaction term of control deficit × provocation was significant in predicting play through pain and injury (Β = 0.10 **, *p* < 0.05). The inclusion of control deficit × provocation interaction increased the predictive power of the main effect by 0.01 percent (R squared change = 0.01, F Change = 5.15 **, *p* < 0.05) from 0.24 percent to 0.25 percent (See Figure 4).

**Figure 5:**
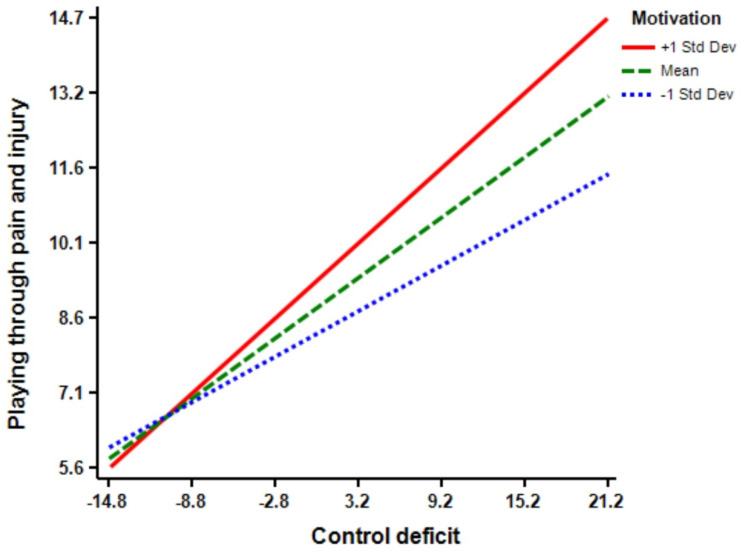
Motivation by control balance deficit interaction.

**Figure 6:**
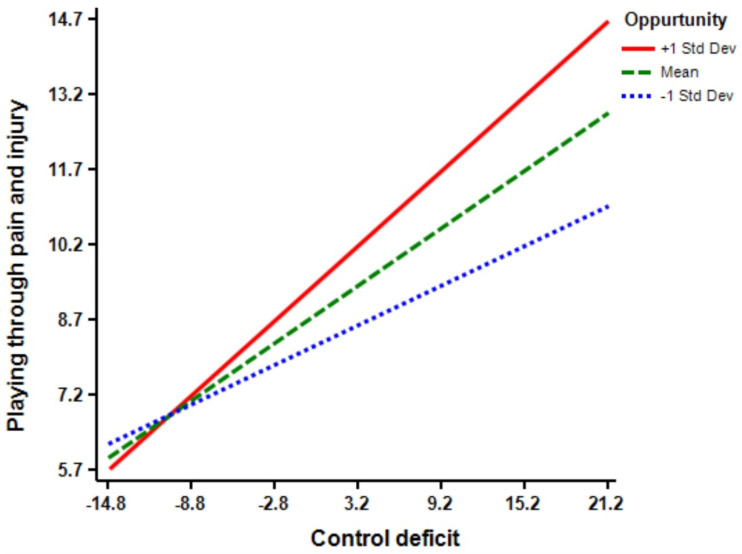
Opportunity by control balance deficit interaction.

**Figure 7:**
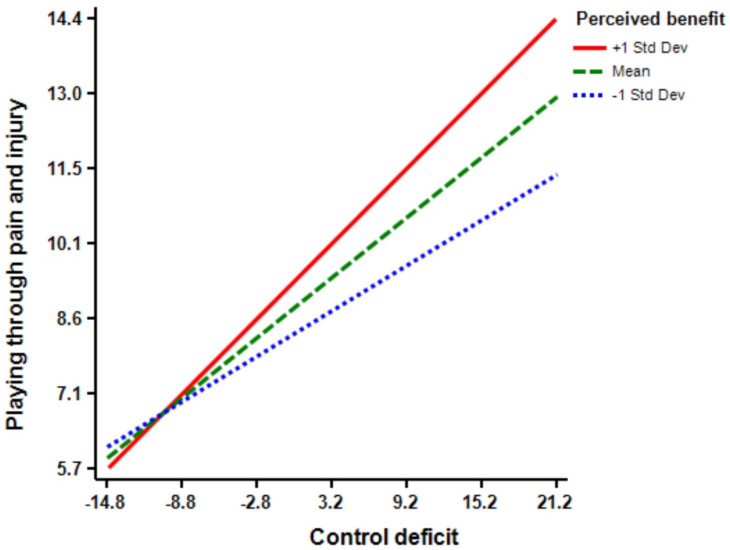
Perceived benefit by control balance deficit interaction.

The regression model (Model 5) shows that motivation (Β = 0.16 *, *p* < 0.01) has a significant effect on athletes’ playing through pain and injury. Additionally, the regression model (Model 6) indicates that the interaction term of control deficit × motivation is predictive of playing through pain and injury (Β = 0.12 **, *p* < 0.01). The inclusion of control deficit × motivation interaction increased the predictive power of the main effect by 0.02 percent (R squared change = 0.02, F Change = 7.92 **, *p* < 0.01) from 0.24 percent to 0.26 percent (See Figure 5). Moreover, the regression model (Model 7) demonstrates that opportunity (Β = 0.18 **, *p* < 0.01) has a significant effect on athletes’ playing through pain and injury. Model 8 shows that the interaction term of control deficit × opportunity is significant in predicting playing through pain and injury (Β = 0.14 **, *p* < 0.01). The inclusion of control deficit × opportunity interaction increased the predictive power of the main effect by 0.02 percent (R squared change = 0.02, F Change = 10.21 **, *p* < 0.01) from 0.25 percent to 0.27 percent (See Figure 6). According to Model 9, perceived benefit (Β = 0.15 **, *p* < 0.01) has a significant effect on athletes’ playing through pain and injury. Model 10 shows that the interaction terms of control deficit × perceived benefit is significant (Β = 0.12 **, *p* < 0.01), and the inclusion of control deficit × perceived benefit interaction increased the predictive power of the main effect by 0.01 percent (R squared change = 0.01, F Change = 7.41 **, *p* < 0.01) from 0.24 percent to 0.25 percent (See Figure 7). Finally, Model 11 suggests that, although the inclusion of all interaction terms in the full model increased the predictive power of the main effect by 0.02 percent, but the effect is not significant (R squared change = 0.03, F Change = 1.90).

Table 4 exhibits the results for the slope of the regression line test. Briefly, the findings indicate that simple slopes for the association between all moderators and playing through pain and injury for low (−1 SD below the mean), moderate (mean), and high (+1 SD above the mean) levels were significant.

## 6. Discussion

Playing through pain and injury can pose serious mental and physical risks to athletes by increasing the likelihood of sustaining various chronic injuries [41,42]. Based on Tittle’s [10,11] control balance theory, we argued that athletes’ deviant and risky behaviors are byproducts of their control deficits in sports and social settings. High levels of control deficit can motivate athletes to extend his/her control in the sports context. One possible way to restore control may involve playing with pain and injury. Tittle also contended that several major contingencies should be considered to understand the impact of control imbalance on deviant behavior; he posited that low self-control, opportunities, situational provocations, and perceived benefits could condition the relationship between perceived control deficit and involvement in risky behaviors. Our study revealed two key findings.

First, control deficit is significantly and positively associated with playing through pain and injury across all models. Our findings provide strong support for Tittle’s [11] core proposition that control imbalance can result in risky behaviors. Specifically, athletes who perceive an insufficient amount of control over different life domains, including family and sports, are more likely to play through pain and injury. These results suggest that athletes may choose to restore their control when they suffer from a control deficit and that they could rely on risky behaviors in an attempt to gain control over the realm of their sport. Our findings are in line with those of other empirical tests of control balance theory [17,19,24,26].

Second, all five interactions derived from the control balance theory are significant in each moderator model. These findings illuminate the importance of considering the contingencies theorized by Tittle [10,11]. Control deficit interacts with provocation in predicting playing through pain and injury in the positive direction, meaning that when athletes suffering from control deficit are provoked (e.g., through unstable team positions), they are more likely to play through pain and injury. The multiplicative interaction term between control deficit and opportunity is significant, suggesting that the impact of control deficit on playing through pain and injury is stronger when more opportunities (e.g., coaches’ positive beliefs about playing with pain and injuries) present themselves. Finally, control deficit and perceived benefit interact to predict playing through pain and injury. The effect of control deficit on playing through pain and injury is stronger at moderate or high levels of perceived benefits (e.g., it would advance my sports career if I accept and tolerate pain and injury). Our findings are congruent with both the major tenets of control balance theory and previous studies investigating the conditioning effects of contingencies [25,27]. Nonetheless, our results are inconsistent with some research reporting the significant conditional effect of self-control [16,17].

Our study highlights that control deficit can lead professional soccer players to play through pain and injury, which, in turn, exposes them to higher psychological, physiological, and physical health risks [32,41]. Although playing through pain and injury can bring several advantages to athletes by demonstrating high levels of commitment, loyalty, and sacrifice to teammates, coaches, and fans, the potentially damaging dimensions of playing through pain and injury should not be underestimated [43]. Our results reflect but one study. However, if more research replicates our findings, it will be important for sports policy initiatives to incorporate attempts to change the sports subculture and provide more opportunities for athletes to exercise control over their sports domain. For example, athletes and support resources could work together to create alternative definitions of athletic accomplishment and health other than performance-focused success. Considering the importance of the athlete’s identity (e.g., sacrificing for the game and playing through injury and pain), redefining values and ethics and promoting them will be necessary to stop athletes from suffering from control deficits. This effort should be accompanied by changing the sport’s structure that ostracizes athletes who do not accept risks and play through pain and injury. If athletes recognize that accepting reasonable limits to prove their sports commitment is important and critical to healthy sports culture and the members of the sporting community, they will not necessarily consider the acceptance of physical risks as demonstrating strong character and reaffirming their athlete identity. Finally, practitioners may consider assessing the levels of control deficit among athletes and screening for health-compromising behaviors so that their behavioral tendencies do not result in more severe and chronic injuries.

### Limitations

Although our application of Tittle’s [10,11] control balance theory can shed light on why athletes accept the risk of injury and play through pain, several limitations of the study should be acknowledged. These limitations may offer future research opportunities to examine the sources of playing through pain and injury among athletes. First, our data were cross-sectional, which cannot address the temporal order between variables of interest examined in this study. Second, our findings’ external validity should be qualified because we used data from a sample of athletes from the Guilan province of Iran. Third, our measure of key variables may not be ideal. Although critical components in the current study have been operationalized based on previous research, we had to revise them to be more relevant to our dependent variable, which is not defined in the same way as traditional deviance. There remains a need to replicate our study with different measures to ensure the validity of our findings. Finally, we relied on data from a single source, presenting the potential bias associated with spurious internal consistency. Therefore, future researchers can derive independent and dependent variables using independent data sources. For example, key control balance theory components can be obtained from self-reported data, but behavioral tendencies toward playing with injury can come from official records.

## 7. Conclusions

All told, although injury-related behavioral tendencies among athletes have been viewed as part of sports culture [35,44], athletes who frequently play through pain and injury are more likely to experience the consequences of taking serious mental and physical risks [41]. Although early efforts to study the phenomenon have addressed important questions (e.g., the normalization process of sports injury and the importance of sports-related networks), there is still much about play through pain and injury yet to be explored. This is especially the case regarding athletes′ role as agents of control, and our findings underscore this point. That is, we tested to discover whether the control deficit from which athletes suffer can predict behavioral tendencies toward play through pain and injury. The results generally reinforced the utility of Tittle’s [10,11] control balance theory. Our findings suggest that athletes’ role as agents of control should continue to be the subject of self-directed violence research.

## Figures and Tables

**Table 1:** Validity and reliability of research measurement instruments.

	Factor Loadings	AVE	CR	α
	Minimum–Maximum			
Low self-control	0.76–0.88	0.67	0.92	0.91
General control deficit	0.72–0.84	0.58	0.89	0.90
Sport control deficit	0.62–0.75	0.51	0.88	0.87
Opportunity	0.82–0.82	0.67	0.86	0.88
Motivation	0.80–0.87	0.69	0.90	0.91
Perceived benefits	0.75–0.93	0.66	0.89	0.88
Provocation	0.72–0.81	0.59	0.85	0.83
Playing through pain and injury	0.81–0.88	0.73	0.89	0.87

Note. AVE = average variance extracted; CR = composite reliability.

**Table 2:** Zero-order correlations between independent and dependent variables (*n* = 749).

	M	SD	1	2	3	4	5	6	7
1. Playing through pain and injury	8.86	3.46	1.00						
2. General control deficit	13.26	3.97	0.40 **	1.00					
3. Sport control deficit	14.52	4.40	0.41 **	0.47 **	1.00				
4. Low self-control	16.11	5.68	0.39 **	0.37 **	0.29 **	1.00			
5. Provocations	11.64	3.88	0.17 **	0.09 *	0.03**	0.12 **	1.00		
6. Motivation	11.01	3.92	0.26 **	0.21 **	0.19 **	0.15 **	0.08 *	1.00	
7. Opportunity	8.20	2.84	0.31 **	0.27 **	0.26 **	0.25 **	0.10 *	0.31 **	1.00
8. Perceived benefit	11.60	4.01	0.25 **	0.23 **	0.18 **	0.38 **	0.11 *	0.23 **	0.30 **

Note. * *p* < 0.05. ** *p* < 0.01.

**Table 3:** Results for interaction analyses predicting athletes’ playing through pain and injury (*n* = 410).

	Low Self-Control		Provocation		Motivation		Opportunity		Perceived Benefit		Full Model	
	Model 1	Model 2	Model 3	Model 4	Model 5	Model 6	Model 7	Model 8	Model 9	Model 10	Model 11	Model 12
Control deficit	0.38 **	0.36 **	0.46 **	0.42 **	0.43 **	0.42 **	0.41 **	0.40 **	0.44 **	0.41 **	0.32 **	0.29 **
Low Self-Control	0.24 **	0.25 **									0.20 **	0.19 **
Provocation			0.14 **	0.15 **							0.10 *	0.09 *
Motivation					0.16 **	0.15 **					0.11 *	0.10 *
Opportunity							0.18 **	0.17 **			0.10 *	0.10 *
Perceived benefit									0.15 **	0.14 **	0.03	0.03
Control deficit × Low Self-Control		0.11 **										0.02
Control deficit × Provocation				0.10 *								0.06
Control deficit × Motivation						0.12 **						0.04
Control deficit × Opportunity								0.14**				0.03
Control deficit × Perceived benefit										0.12 **		0.05
R squared	0.27	0.28	0.24	0.25	0.24	0.26	0.25	0.27	0.24	0.25	0.31	0.33
R squared change	0.27	0.01	0.24	0.01	0.24	0.02	0.25	0.02	0.24	0.01	0.31	0.02
F Change	75.27 **	6.92 **	63.94 **	5.15 *	65.74 **	7.92 **	67.33 **	10.21 **	64.36 **	7.41 **	30.41 **	1.90

Note. Beta coefficient represented in table, * *p* < 0.05., ** *p* < 0.01.

**Table 4:** Results for Slope of Regression Line Tests (*n* = 410).

	Low Self-Control		Provocation		Motivation		Opportunity		Perceived Benefit	
	b (SE)	t	b (SE)	t	b (SE)	t	b (SE)	t	b (SE)	t
Low (−1 SD below the mean)	0.12 ** (0.03)	4.31	0.15 ** (0.03)	4.47	0.15 ** (0.03)	5.39	0.13 ** (0.03)	4.36	0.15 ** (0.03)	4.70
Moderate (mean)	0.17 ** (0.02)	7.94	0.20 ** (0.02)	9.22	0.20 ** (0.02)	9.60	0.19 ** (0.02)	8.77	0.20 ** (0.02)	8.92
High (+1 SD above the mean)	0.22 ** (0.03)	8.32	0.25 ** (0.02)	10.23	0.25 ** (0.03)	9.53	0.24 ** (0.02)	9.39	0.24 ** (0.02)	9.85

Note. ** *p* < 0.01.

## Data Availability

The data presented in this study are not publicly available due to confidential issues of the participants.
